# Viability droplet digital polymerase chain reaction accurately enumerates probiotics and provides insight into damage experienced during storage

**DOI:** 10.3389/fmicb.2022.966264

**Published:** 2022-10-27

**Authors:** Anthony Kiefer, Phillip M. Byrd, Peipei Tang, Gregory Jones, Kevin Galles, Vincenzo Fallico, Connie Wong

**Affiliations:** IFF Health and Biosciences, Danisco USA, Inc., Madison, WI, United States

**Keywords:** probiotic, storage, v-ddPCR, viability, enumeration, PMA, EMA, PE51

## Abstract

Probiotics are typically enumerated by agar plate counting (PC) techniques. PC has several limitations including poor specificity, high variability, inability to enumerate dead cells, viable but non-culturable cells and cells in complex matrices. Viability droplet digital polymerase chain reaction (v-ddPCR) is an emerging enumeration technique with improved specificity, precision, and the ability to enumerate cells in varying states of culturability or in complex matrices. Good correlation and agreement between v-ddPCR and PC is well documented, but not much research has been published on the comparison when enumerating freeze-dried (FD) probiotics during storage. In this study, v-ddPCR utilizing PE51 (PE51-ddPCR), a combination of propidium monoazide (PMA) and ethidium monoazide (EMA), was evaluated as alternative enumeration technique to PC on blends of four FD probiotic strains over the course of a 3-month storage study with accelerated conditions. When PMA and EMA are combined (PE51), this study demonstrates agreement (bias = 7.63e+9, LOA = 4.38e+10 to 5.9e+10) and association (*r* = 0.762) between PC and v-ddPCR, at or above levels of an accepted alternative method. Additionally, v-ddPCR with individual dyes PMA and EMA provide insight into how they individually contribute to the viable counts obtained by PE51-ddPCR and provide a more specific physiological understanding of how probiotics cope with or experience damage during storage.

## Introduction

Probiotics are defined as “live microorganisms that, when administered in adequate amounts, confer a health benefit on the host” (FAO/WHO, 2002; Hill et al., 2014). Two well studied probiotic genera are *Lactobacillus* and *Bifidobacterium.* Recently, the genus *Lactobacillus* was subject to a reclassification that created 25 separate genera and reclassified several species of common probiotics into new genera such as *Lacticaseibacillus, Lactipantibacillus, Limosilactobacillus, Ligilactobacillus, Levilactobacillus, Lentilactobacillus* and *Latilactobacillus* among others (Zheng et al., 2020). Live probiotic *Lactobacillus* and *Bifidobacterium* strains have been demonstrated to provide immune (Paineau et al., 2008; Leyer et al., 2009; Moens et al., 2019), digestive (Ringel-Kulka et al., 2011; Favretto et al., 2013; Eskesen et al., 2015) and even cognitive (Stenman et al., 2020) health benefits, among others, when taken in the correct amount (dosage).

Agar plate count (PC) is the current standard for enumerating live probiotic cells and confirming the presence of health-promoting dosages in commercial products. While these methods have a successful and well documented history, they also have demonstrated difficulty enumerating probiotics under certain conditions. PC cannot enumerate dormant cells, which are viable and have the potential to provide probiotic effects but are not in a culturable state (VBNC; Panutdaporn et al., 2006; Lahtinen et al., 2008; Pawlowski et al., 2011; Ramamurthy et al., 2014; Zhao et al., 2021). PC also lacks the ability to enumerate injured/dead microbes (Parabiotics/Postbiotics; Barros et al., 2020; Salminen et al., 2021). While selective PC methods exist, they are not capable of distinguishing bacteria at the strain level and can often have difficulty distinguishing even at the species level (Iwana et al., 1993; Van de Casteele et al., 2006). This presents a regulatory concern for multi-strain probiotic products with well documented health benefits and clinical outcomes (Bibiloni et al., 2005; Lahtinen et al., 2008; McFarland et al., 2016). Additionally, PC encounters issues when enumerating probiotics in complex matrices such as intestinal and fecal samples where large numbers and types of other microorganisms persist (Jackson et al., 2002).

Alternative methods including flow cytometry, quantitative PCR, chip digital PCR (cdPCR), droplet digital PCR (ddPCR) and oil-enveloped ddPCR have been proposed to replace or even supplement agar PC methods. These methods, often termed “rapid methods” can reduce time to results, offer increased specificity, improved precision and are capable of enumerating bacteria in complex matrices (Herbel et al., 2013; Sattler et al., 2014; Gobert et al., 2018; Roussel et al., 2018; Hansen et al., 2020; Zhao et al., 2022). These rapid methods take different approaches to determine viability, generally using viability dyes or treatments. Viability dyes distinguish viable cells from dead cells by targeting cellular functions such as membrane potential, membrane integrity and cellular activity.

Propidium monoazide (PMA) and ethidium monoazide (EMA), photo-reactive DNA binding dyes which are well documented for their use in viability ddPCR (v-ddPCR) individually and in combination (PE51, PEMAX; Gobert et al., 2018; Hansen et al., 2020; Kiefer et al., 2020). PMA and EMA covalently bind DNA of dead or damaged cells, thus preventing their amplification by PCR. PMA is excluded from living cells because it cannot pass through intact cell membranes. EMA can pass through the cell membrane but is excluded from the cell by active efflux pump activity (Nocker et al., 2006; Codony et al., 2015; Baymiev et al., 2020). While previous studies have demonstrated agreement between v-ddPCR methods and PC on freeze-dried (FD) probiotics (Hansen et al., 2020; Kiefer et al., 2020), little research has been conducted to assess this agreement on commercially formulated FD probiotic products during storage.

Loss in viable probiotic count is known to occur even under ideal storage conditions. The International Scientific Association for Probiotics and Prebiotics (ISAPP) has asserted that a probiotic must be present, in the correct dosage until the end of the product shelf life (Hill et al., 2014). To ensure this criterion is met, probiotic products undergo extensive stability testing during storage. Traditional stability studies take years to complete and are labor intensive. Increased water activity (Aw), the amount of water in a sample that is available to react, has been documented to decrease the viability of FD probiotics during storage and has been used previously to accelerate storage conditions (Abe et al., 2009; Celik and O’Sullivan, 2013; Albadran et al., 2015).

In this study, three v-ddPCR methods were analyzed for linearity using four FD probiotic strains. Next, short-term and accelerated (increased Aw) storage studies were performed using FD probiotic blends under three different storage conditions. Stored probiotic blends were assessed for DNA stability *via* ddPCR without viability treatment (t-ddPCR) as well as probiotic viability (stability) by v-ddPCR with PE51 (PE51-ddPCR) and traditional agar PC. PE51-ddPCR was evaluated as an alternative method to PC by analyzing viability results for agreement and association. Lastly, PMA-ddPCR and EMA-ddPCR were also performed on stored probiotic blends to better understand their individual contributions to the viable counts obtained by PE51-ddPCR and to provide more specific physiological characterization.

## Materials and methods

### Probiotic formulations and treatments

Microcrystalline cellulose (MCC) with two different levels of Aw (ambient or high) was prepared as follows. One aliquot (1 kg) was left unaltered (ambient Aw), whereas another (high Aw) was loaded into a sterile stainless-steel tray. Tray was covered with plastic and vented with ~8 evenly dispersed 1-inch crosswise (X) slits to allow moisture ingress. Tray was held in an environmental chamber at 30°C and 75% relative humidity (RH; Caron, Marietta, OH, United States) for 9.5 h. Tray was intermittently removed to homogenize by manual hand mixing with sterile scoop and to sample for Aw. Initial Aw reading was 0.016, after 9.5 h of treatment an ending Aw of 0.202 was achieved. Aw was measured on HygroLab C1 device per manufacturers recommendations (Rotronic, Hauppauge, New York, United States).

Four probiotic strains were used in this study, *Bifidobacterium animalis* subsp. *lactis* Bi-07 and Bl-04, *Lactobacillus acidophilus* La-14 and *Lacticaseibacillus rhamnosus* Lr-32. FD probiotics were blended with ambient (short-term) and high (accelerated) Aw MCC in appropriate amounts to make 250 g (each) of material with a final concentration of ~7.94e+10 CFU/g which is the equivalent of a 5 billion CFU dosage in size zero capsules (Lonza Capsugel, Morristown, New Jersey, United States).

Blends were partitioned in 20 g aliquots and sealed in oxygen and moisture barrier foil sachets to minimize changes in Aw. Sachets were placed under three storage conditions: 4°C and uncontrolled relative humidity (RH) (4C), 25°C and 60% RH (25ICH), and 30°C and 65% RH (30ICH) per International Conference on Harmonisation (2003). Remaining material was used for time zero (T0) analyses. Blends were analyzed after 1, 2 and 3 months of storage.

### Plate count enumeration

Traditional agar PC methods were utilized as previously outlined by Hansen et al. (2020), and in accordance with United States Pharmacopeia (USP) monographs for *Bifidobacterium animalis* subsp*. lactis* (USP-NF, 2017a), *Lactobacillus acidophilus* La-14 (USP-NF, 2017b), and *Lacticaseibacillus rhamnosus* (USP-NF, 2021). To summarize, 1 g of powder, per blend, was weighed into Whirl-Pak sample bags (Whirl-Pak, Madison, WI, United States) in triplicate. Samples were rehydrated in 99 g of De Man, Rogosa and Sharpe (MRS) broth (BD, Franklin Lakes, NJ, United States), blended using Stomacher 400 Circulator (Seward, Worthing, West Sussex, UK) at 230 RPM for either 30 s (Bi-07, Bl-04) or 2 min (La-14, Lr-32) then held for 30 min at room temperature. After 30 min, samples were blended once more, as previously specified.

Samples were then serially diluted in 99 ml Flip-Top Dilution Bottles with Peptone Water (3 M, Maplewood, MN, United States) to a target concentration between 25 and 250 CFU/ml. One ml of each diluted sample was pipetted onto three empty petri dishes. Approximately 15 ml of 45°C MRS agar (BD, Franklin Lakes, NJ, United States; Lr-32) or MRS agar with 0.05% cysteine (Sigma-Aldrich, St. Louis, MO, United States; Bi-07, Bl-04, La-14) was added to each plate, swirled to mix, then left to solidify. After solidification, plates were placed in anaerobic jars with GasPak EZ sachets (BD) and incubated at 37°C for 48–72 h. After incubation, resulting colonies were counted and results were reported in CFU/g after accounting for dilution factors.

### Droplet digital polymerase chain reaction

Primer and probe assays used for ddPCR enumeration of Bi-07, Bl-04, and La-14 samples were previously reported (Hansen et al., 2018, 2020). The primer and probe assay for ddPCR enumeration of Lr-32 was designed for this study and targets a gene that encodes a hypothetical protein. All assays utilized in this study target single copy genes.

ddPCR methods were carried out as previously outlined in Kiefer et al. (2020), with some modifications. Briefly, 1 g of powder per blended sample was weighed into a Whirl-Pak in triplicate. Blended samples were rehydrated in 99 ml of Remel Butterfield’s Phosphate Buffer (BPB; Fisher, Hampton, NH, United States). Storage study samples were further diluted in BPB to a target concentration of between 200 and 2000 Copies/μl in the PCR mix. Dilutions ranged from 1:1,000 to 1:20,000 of the initial concentration, depending on the loss of viability in the sample being analyzed. To assess linearity, FD samples were diluted in BPB to an initial concentration equivalent to 2000 Copies/μl in the PCR mix. The initial solution was then diluted over a 2-log concentration gradient prior to viability treatment. Targeted concentrations were 2,000, 1,980, 1,800, 1,500, 1,000, 500, 200, 20 and 0 Copies/g.

For samples undergoing viability treatment (v-ddPCR), 1.2 ml was added to 1.5 ml centrifuge vials and treated with dye (Table 1). For PE51-ddPCR, samples were treated with concentrations of PE51 which were optimized (prior to blending) for agreement with PC (data not shown). PE51 was created by combining PMA and EMA at a molar ratio of 5:1 as established by Codony et al. (2015) to achieve a final dye concentration of 50 μM. For PMA-ddPCR or EMA-ddPCR, PMA and EMA solutions were created at a concentration equal to their individual concentrations in PE51 (41.7 μM PMA, 8.3 μM EMA) each dye was added at the same volume of PE51 (Table 1). After addition of dye, samples were gently vortexed to mix, incubated at 37°C, protected from light and shaken at 200RPM for 30 min to facilitate reaction. After incubation, samples were transferred to PMA-lite LED Photolysis Device (Biotium, Fremont, CA, United States) for 15 min to cross-link dyes and halt further reaction. No viability dye was added to samples to be analyzed for total DNA count (t-ddPCR).

**Table 1:** Viability treatment information for v-ddPCR assays.

Strain	μl of Dye^a^	[PE51]^b^	[PMA]^b^	[EMA]^b^
Bi-07	4	170	140	30
Bl-04	22.5	920	770	150
La-14	32.5	1,300	1,100	200
Lr-32	32	1,300	1,100	200

a Actual volume of dye added to reaction.

b Final concentration of the dye (nM).

One ml of t-ddPCR and v-ddPCR samples was transferred into prefilled 2.0-ml tubes containing Triple-Pure high-impact 0.1-mm zirconium beads (Benchmark Scientific, Edison, NJ, United States). Cells were lysed at 6.30 m/s in Bead Ruptor Elite (Omni International, Kennesaw GA, United States) for an optimized time (1 min for Bi-07, La-14 or 2 min for Bl-04, Lr-32) then placed on ice for ~5 min.

PCR reaction mixtures were created by combining the following reagents in the specified volumes and [concentrations]: 1.68 μl of molecular biology grade water, 18 μl each of forward and reverse primers [5 μM], 8.32 μl of probe [3 μM] (Integrated DNA Technologies, Coralville, IA, United States; Table 2), 50 μl of ddPCR Supermix for probes (no dUTP) [2X] and 4 μl of lysed sample. For stored blends with low viable counts, the following changes were made to the mixture: 0.90 μl each of forward and reverse primers [100 μM], 8.32 μl of probe [3 μM] (Table 2), 50 μl of ddPCR Supermix for probes (no dUTP) [2X] and 40 μl of lysed sample (addition of molecular biology grade water was not required). Lysed sample from a dilution of 1:2,000 in BPB thus creating a 10× increase in copies in the PCR mix.

**Table 2:** Primer and probe sequences for ddPCR assays.

Strain	Oligo type	Sequence (5′–3′)
Bi-07	Forward	TTC AAG CCG ACG TAC TTG CT
	Probe	/5HEX/TC GCC AAT G/ZEN/C CGT CGA CCA T/3IABkFQ/
	Reverse	TGA TTC GCA TCA TCG GTC CC
Bl-04	Forward	CTT CCC AGA AGG CCG GGT
	Probe	/56-FAM/CG AAG ATG A/ZEN/T GTC GGA ACA CAA ACA CCC GG/3IABkFQ/
	Reverse	CGA GGC CAC GGT GCT CAT ATA GA
La-14	Forward	CCG GTT AAT AAA ATC TTT TCA CCT TG
	Probe	/56-FAM/AG TTG ATC A/ZEN/G TCA GCA AGT AGT GTT ATG G/3IABkFQ/
	Reverse	GCA GTT ATT AAT CGT GAT TTG CAT ATA AAT T
Lr-32	Forward	GCA GTG GCA TGA CTA CGT
	Probe	/5HEX/AT TAG TGA G/ZEN/C GTT TTT CGG CTT TTT TAG CT/3IABkFQ/
	Reverse	AGA TTT TTT CGC TTG TCT TCT TCT

PCR reaction mixture (25 μl) was added in triplicate to ddPCR 96-Well Plates (Bio-Rad, Pleasanton, CA, United States). Plates were heat sealed by PX1 PCR Plate Sealer with pierceable foil PCR plate seals. Plate was loaded into AutoDG along with pipette tips, AutoDG cartridges, and a new 96-well plate to form droplets with droplet generating oil for probes. Newly formed droplets were heat sealed as previously described and transferred to C1000 touch Thermal Cycler. Thermal cycling conditions: 40 cycles of 95°C for 10 min, 95°C for 30 s, and 56/60°C (ramp rate 2.0°C/s; La-14/others, respectively) for 1 min, followed by 98°C for 10 min, then held at 4°C until reading. After PCR amplification, plate was analyzed on QX200 Droplet Reader with QuantaSoft v.1.7 software. Thresholds for positive droplets were set at 2,000 for Bi-07, Lr-32 and La-14 and 3,000 for Bl-04 on their respective channel (Table 2; channel 1 – FAM, channel 2 – Hex).

### Statistical analysis

**Figure 1:**
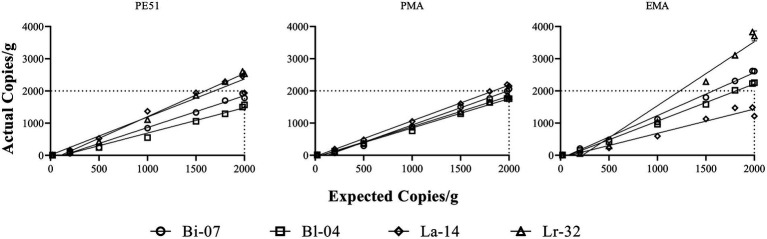
Simple linear regression analysis of Actual Copies/g against Expected Copies/g. Expected Copies/g are hypothetical counts targeted over the 2-log dilution series. Actual Copies/g are the results obtained from v-ddPCR of the dilution series. Correlation coefficient and slope values are reported in Table 3.

**Figure 2:**
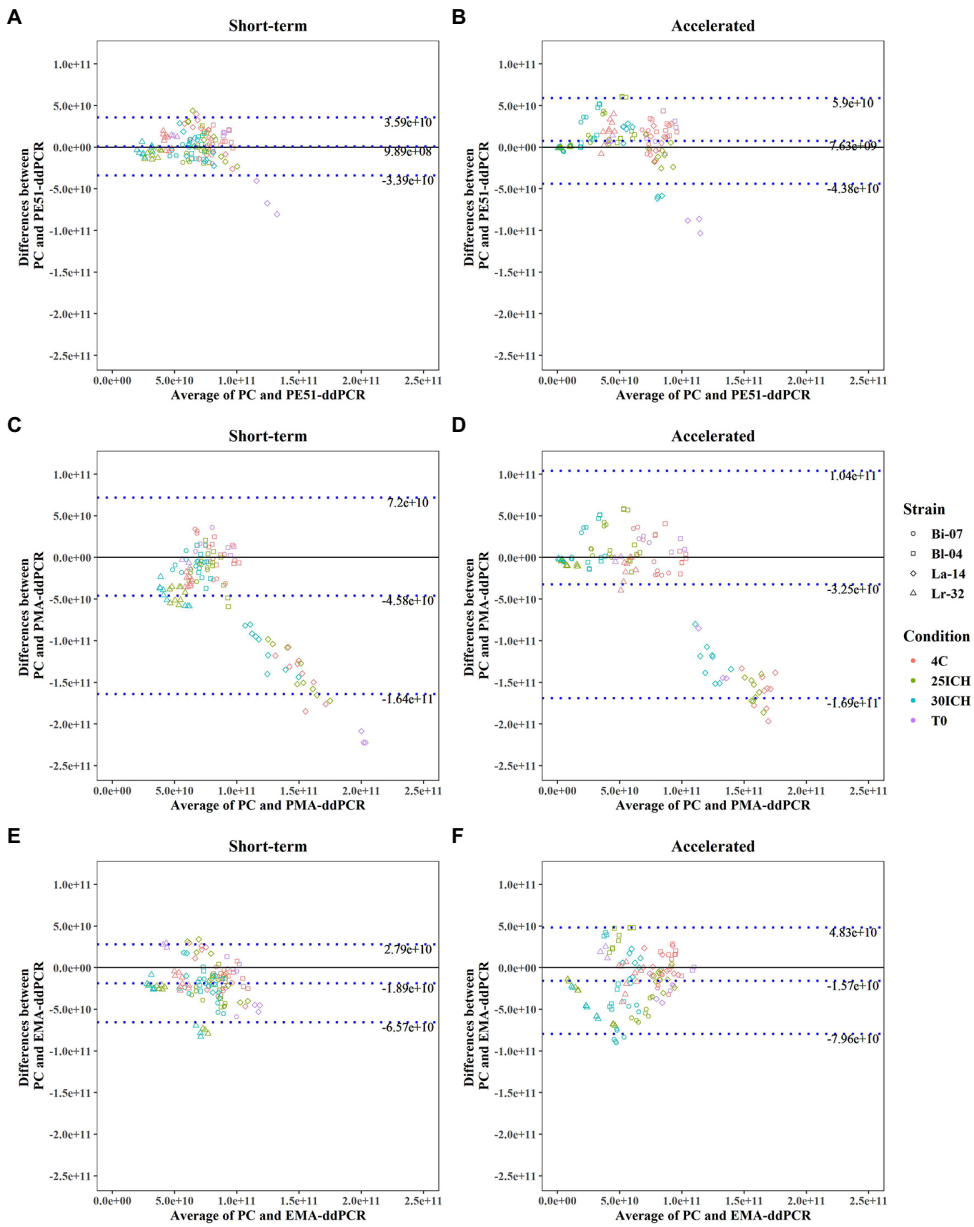
Bland–Altman Analysis between results of PC and v-ddPCR methods on probiotic blends under short-term and accelerated conditions with average difference (bias) and Limits of Agreement (LOA) reported. **(A,B)** PE51-ddPCR vs. PC, **(C,D)** PMA-ddPCR vs. PC, **(E,F)** EMA-ddPCR vs. PC, short-term and accelerated storage conditions, respectively.

**Figure 3:**
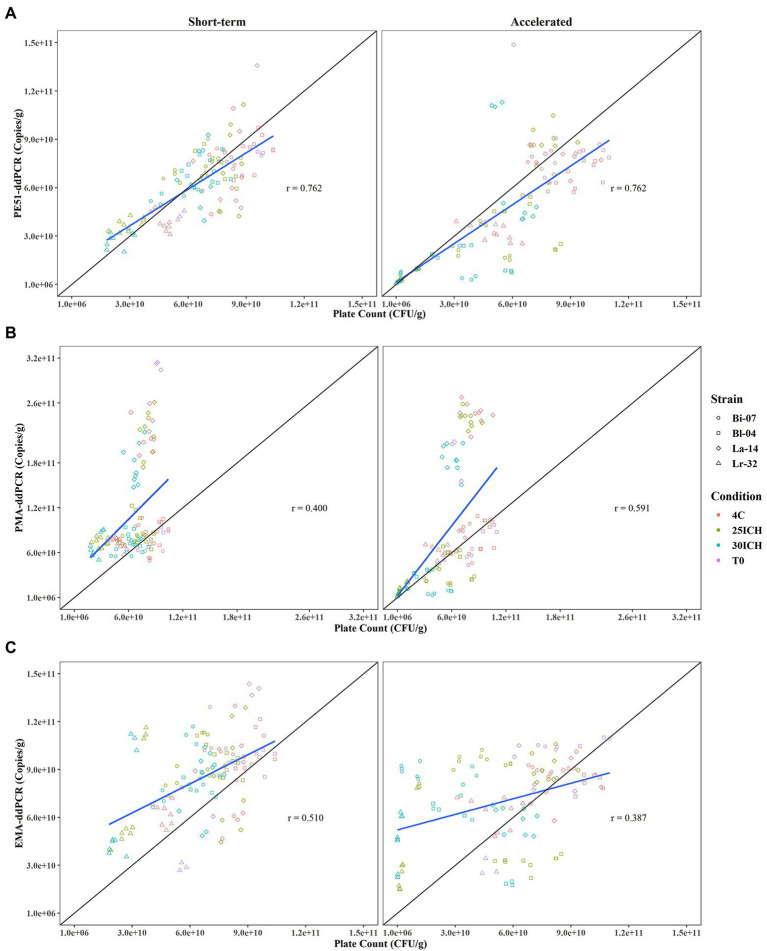
Pearson Correlation analysis comparing viability results of PC and v-ddPCR methods over 3 month short-term and accelerated storage conditions with Correlation Coefficients (*r*) reported. **(A)** PE51-ddPCR vs. PC, **(B)** PMA-ddPCR vs. PC, **(C)** EMA-ddPCR vs. PC for short-term and accelerated storage conditions, respectively.

A simple linear regression analysis was performed on v-ddPCR data of FD probiotics over a 2-log dilution series (Figure 1). Correlation coefficient (*r*) and slope of the regression analyses were reported (Table 3). Bland–Altman analysis was conducted to assess agreement between analytical measurements (Bland and Altman, 1986; Figure 2). Differences (bias) between measurement pairs (Method 1 – Method 2) was plotted against the mean of the paired measurements. The overall matching between the two methods was summarized and evaluated by bias and limits of agreement (LOA). Results obtained by v-ddPCR with PE51, PMA and EMA were compared to PC using Pearson correlation analysis (Figure 3). A linear function was overlaid to visualize their association. Exploratory analysis of PE51-ddPCR, PMA-ddPCR, EMA-ddPCR and PC viability data was performed using Principal Components Analysis (PCA) after standardization of the variables (mean = 0; SD = 1). Figures and analyses for simple linear regression, percent recovery heat map and PCA were generated with GraphPad Prism version 9.2.0 for Windows (GraphPad Software, San Diego, California, United States). Statistical analysis and figure generation for Bland–Altman and Pearson correlation was performed in R v4.1.1 with packages tidyverse v1.3.1, lsmeans v2.30–0, rstatix v0.7.0, ggpubr v0.4.0, scales v1.1.1, RColorBrewer v1.1–2, and knitr v1.33.”

**Table 3:** Correlation coefficient (*r*) and slope results from simple linear regression analysis (Figure 1).

Strain	Bi-07		Bl-04		La-14		Lr-32	
Result	*r*	Slope	*r*	Slope	*r*	Slope	*r*	Slope
PE51	0.99	0.99	0.99	0.79	0.98	1.18	0.99	1.34
PMA	0.99	1.07	1.00	0.91	1.00	1.11	1.00	0.95
EMA	0.99	1.33	0.99	1.16	0.98	0.75	0.98	1.99

## Results

### Linearity of v-ddPCR methods

A linear analysis (Figure 1) showed strong positive correlation between actual and expected counts for all three v-ddPCR methods applied to all four strains tested. A 2-log dilution series prior to treatment with PE51, PMA and EMA were analyzed and correlation coefficients (*r*) ranged between 0.98–1.00. Most slopes were close to 1, demonstrating a 1:1 ratio between expected and actual counts. EMA deviated furthest from this relationship (0.75–1.99), followed by PE51 (0.79–1.34), while PMA deviated least (0.91–1.11).

### Effects of Aw on recovery of viable cells

**Figure 4:**
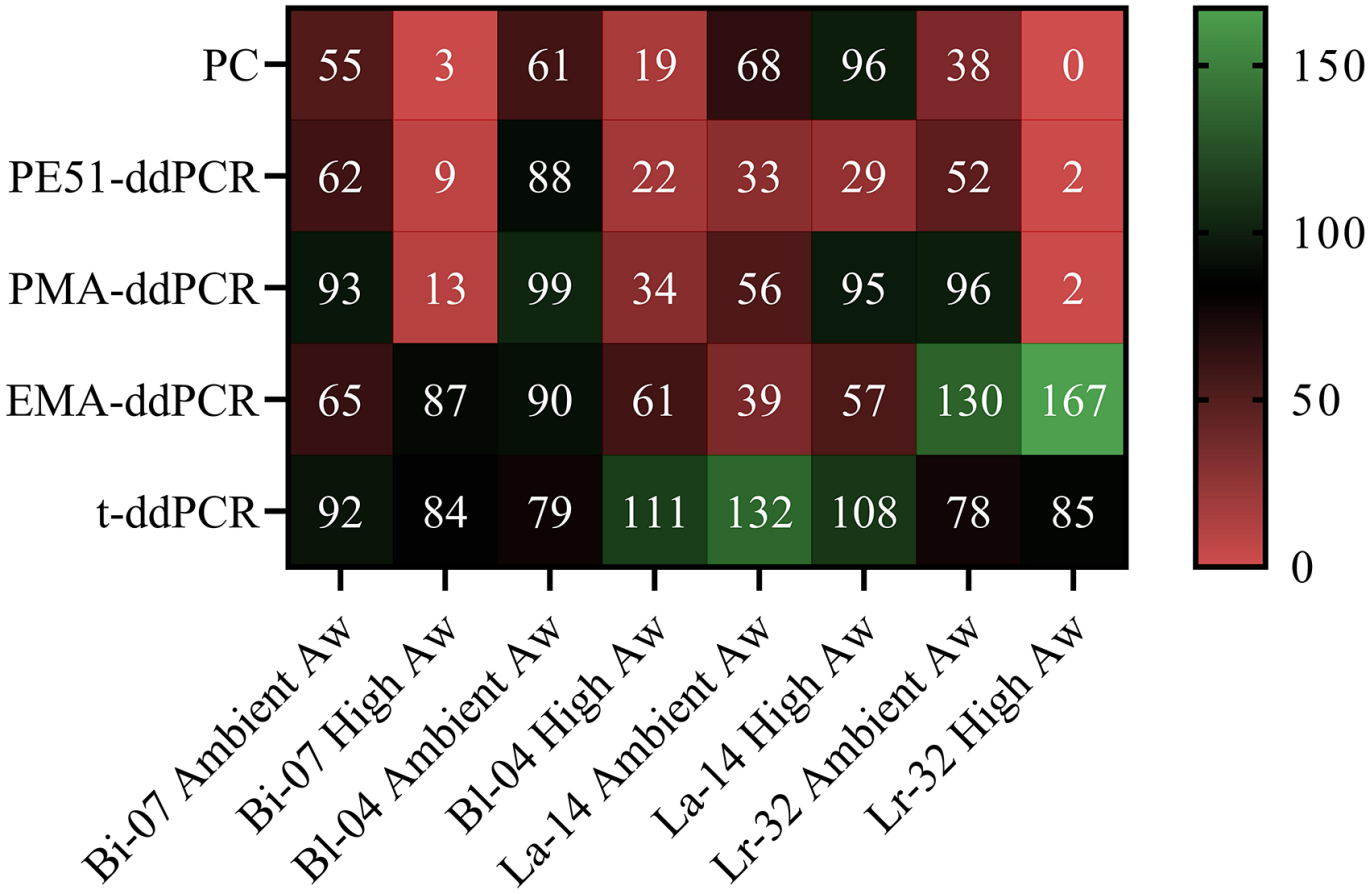
Heat map of PC and v-ddPCR viability results as measured by percent recovery (T3/T0) of probiotic blends after short-term and accelerated storage for 3 months at 30ICH.

DNA was found to be relatively stable, t-ddPCR results remained high for all four strains under all conditions ranging from 78% to 132% with an average percent recovery of 96%. High concentrations of DNA from dead or highly damaged cells were detected. While blends were formulated to a viable concentration of 7.94e+10 CFU/g, T0 t-ddPCR counts averaged 3.37e+11 CFU/g (Bi-07 = 1.75e+11 CFU/g, Bl-04 = 3.05e+11 CFU/g, La-14 = 4.89e+11 CFU/g, Lr-32 = 3.77e+11 CFU/g). Percent recovery, or the proportion of the final count in relation to the initial count (T3 √ T0), was calculated for PC and v-ddPCR methods for the most stringent storage condition (3 months at 30ICH; Figure 4). Percent recovery results demonstrated the impact of artificially increasing Aw on PC when comparing short-term and accelerated storage conditions (Bi-07 = 55 vs. 3%, Bl-04 = 61 vs. 19%, La-14 = 68 vs. 96%, Lr-32 = 38 vs. 0%). In agreement, PE51-ddPCR and PMA-ddPCR showed lower percent recovery on samples stored under accelerated conditions for 3 of the 4 strains analyzed. Exceptions were noted in La-14 across all methods where stability tended to be higher in accelerated conditions when compared to short-term conditions. Aw remained consistent throughout the study (Supplementary Data).

### Method agreement between PC and v-ddPCR methods

Bland–Altman analyses, commonly used to assess agreement between two quantitative methods, were applied to v-ddPCR and PC enumeration data (Giavarina, 2015). Analysis revealed that using PE51 resulted in the smallest bias (9.89e+8, 7.63e+9) when analyzing the average difference between PC and v-ddPCR on short-term and accelerated storage samples, respectively (Figure 2). Using PE51 also produced the narrowest 95% LOA for both short-term (−3.39e+10 to 3.59e+10) and accelerated (4.38e+10 to 5.9e+10) conditions. This was followed by the use of EMA with a bias of −1.89e +10 and −1.57e+10 and PMA with a bias of −4.58e+10 and −3.25e+10 for short-term and accelerated conditions, respectively. Additionally, use of either EMA or PMA exhibited higher LOA than PE51 in both conditions.

### Association between PC and v-ddPCR methods

Enumeration data was plotted and analyzed for association by Pearson correlation, a measure of the strength of the linear relationship between two variables, or in this study, two methods (Giavarina, 2015; Figure 3). Results from v-ddPCR and PC methods revealed that PE51-ddPCR had the highest correlation coefficient for short-term and accelerated conditions (*r* = 0.762, *r* = 0.762) when compared to those of PMA-ddPCR (*r* = 0.400, *r* = 0.591) or EMA-ddPCR (*r* = 0.510, *r* = 0.387, respectively). PE51-ddPCR demonstrated a strong positive linear relationship (*r* > 0.7) with PC. In contrast PMA-ddPCR and EMA-ddPCR exhibited weak linear relationships (*r* < 0.6) with PC. While PE51-ddPCR highly correlated with PC in both short-term and accelerated conditions, PMA-ddPCR exhibited better correlation with PC in accelerated conditions and EMA-ddPCR correlated better in short-term conditions.

### Exploratory analysis of single and dual staining v-ddPCR methods

**Figure 5:**
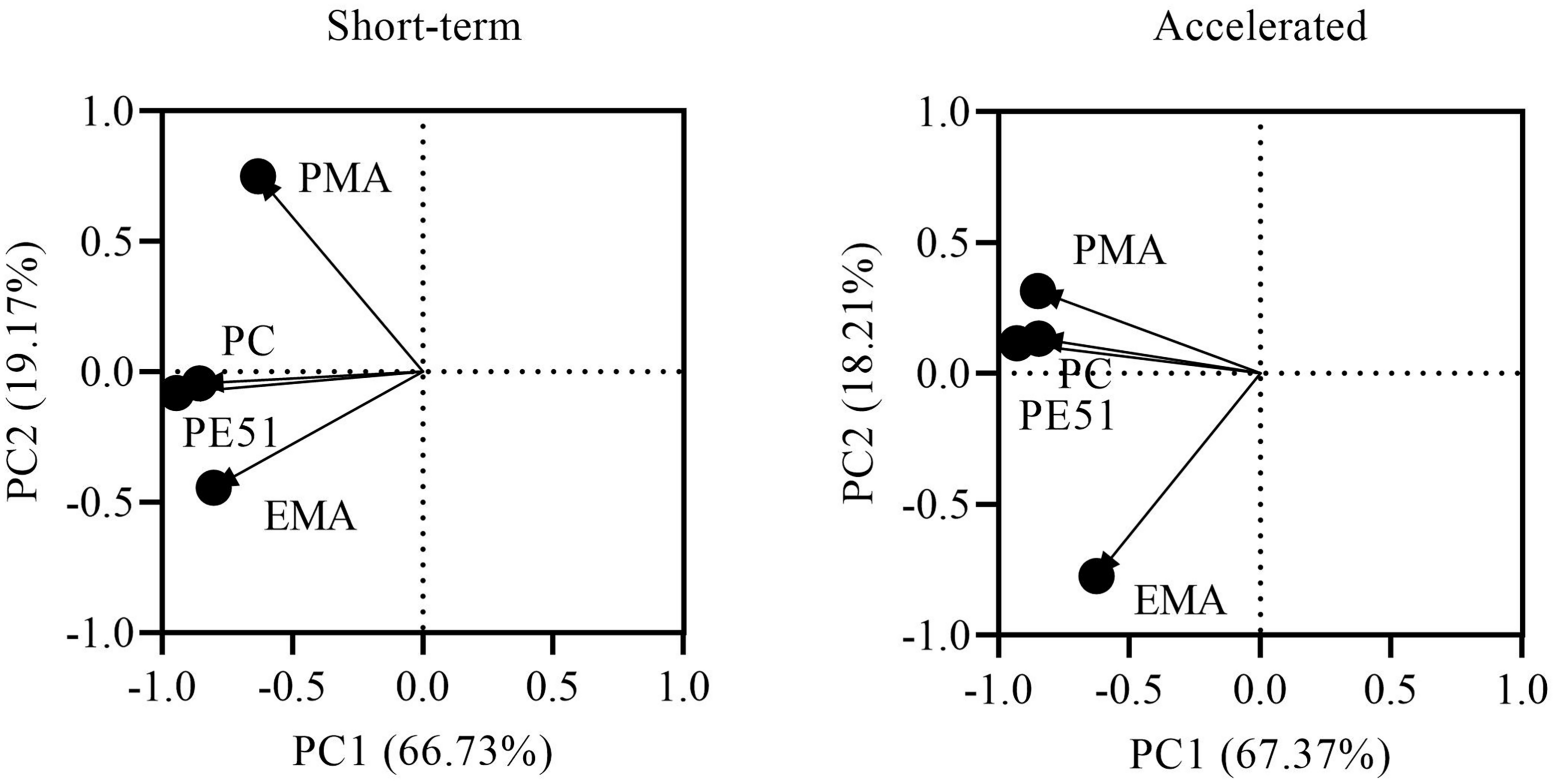
PCA biplot of PC and v-ddPCR methods based on viability results of FD probiotics stored for 3 months under short-term and accelerated conditions.

Exploratory analysis was performed on viability data to evaluate the contributions of individual dye techniques as compared to the dual stain and in relation to PC in short-term and accelerated conditions (Figure 5). The first two principle components were chosen as they explained most of the variation in both the short-term (85.90%) and accelerated (85.58%) data sets. Biplots of short-term and accelerated data grouped all viability assays on the negative side of PC1, demonstrating a close correlation between all methods. However, PC2 grouped EMA-ddPCR and PE51-ddPCR for short-term stored samples while PMA-ddPCR and PE51-ddPCR grouped more closely for accelerated storage samples.

## Discussion

It is well documented that probiotic products lose viability over time resulting in end of shelf life potencies that may fall well below the potency at time of manufacture (Poddar et al., 2014; Arepally et al., 2020). Along with storage temperature and duration, Aw has been demonstrated to be a major contributor to the loss in viability of FD probiotics as measured by traditional agar PC techniques (Abe et al., 2009; Celik and O’Sullivan, 2013). V-ddPCR has been proposed as an improved viability enumeration technique compared to traditional agar PC methods, since it enumerates cells in different physiological states (live, dead) and delivers strain-specific counts with decreased variability (Gobert et al., 2018; Hansen et al., 2020; Kiefer et al., 2020). While v-ddPCR represents the latest development in the enumeration of viable microorganisms, the ability of this method to analyze FD probiotics during storage is not well documented.

Testing probiotics during storage requires a method that can accurately enumerate live cells in the presence of high concentrations of dead or injured cells. In this study, FD probiotics were shown to have high concentration of dead or damaged cells, as indicated by total DNA counts (t-ddPCR) well above that of viable counts (v-ddPCR). Prior to testing stored samples, a linear analysis of v-ddPCR methods was performed on FD probiotic strains over a 2-log dilution series. In agreement with previous studies, PE51-ddPCR and PMA-ddPCR demonstrated a strong linear correlation and 1:1 relationship between expected and actual counts (Truchado et al., 2016; Brosnahan et al., 2019; Kiefer et al., 2020). EMA-ddPCR demonstrated similar trends with three of the four strains, also in agreement with method performance as reported in previous studies (Rudi et al., 2005a,b). An exception was noted for EMA-ddPCR of Lr-32, where a relationship closer to 2:1 indicates a bias to higher concentrations of cells. Overall, these results support the use of v-ddPCR methods on varying concentrations of FD probiotics containing high levels of cells in different states of viability, similar to conditions found in stored probiotics.

To decrease time to results, an accelerated storage study was used in place of a traditional 2-year study. Accelerated conditions were achieved by blending FD probiotics with artificially increased Aw MCC which resulted in viable percent recoveries markedly reduced as compared to short-term storage conditions. This agrees with previous research where this technique was used (Albadran et al., 2015). Conversely, La-14 showed either minimal loss or even increased viability in accelerated conditions for all methods, which may be due to the well documented stability of *Lactobacillus acidophilus* strains (Grosso and Fávaro-Trindade, 2002; Pyar and Peh, 2011). Also, in contrast to other methods, EMA-ddPCR results did not show any discernable trend between short-term and accelerated conditions, suggesting responses to Aw stress were dependent on the strain analyzed. EMA, which distinguishes viable cells by their ability to actively exclude dye through efflux pumps, is often considered a measure of cellular activity (Codony et al., 2015). It has been previously demonstrated that changes in cellular activity, in response to stress, can indeed be strain specific (Morovic et al., 2018).

While the concept of viability is heavily debated, the probiotic industry has a long history of utilizing traditional PC methods for the enumeration of viable probiotics (Kiefer et al., 2020; Wendel, 2022). For this reason, v-ddPCR methods were compared to PC, to assess their ability to accurately enumerate viable probiotics (stability) in short-term and accelerated storage studies. Excellent correlation between PE51-ddPCR and PC (*r* = 0.762) was demonstrated for both short-term and accelerated storage studies. Correlations were found to be at or above what similar studies have deemed a strong positive correlation and at a level indicated as an equivalent method (Naghili et al., 2013; Luedtke and Bosilevac, 2015; Akoglu, 2018; Hansen et al., 2020). Additionally, Bland–Altman analysis of PE51-ddPCR and PC revealed a bias of nearly 2-logs lower than the formulated concentration and LOA centered around zero. These analyses indicate that combining membrane integrity and metabolic activity, as measured by PE51-ddPCR, provide an acceptable rapid method of determining probiotic viability. Small differences found are at a level that would not have major commercial relevance.

Although the dual dye PE51-ddPCR method was demonstrated to accurately enumerate viable cells by combining elements of membrane integrity and efflux pump activity, there is also value in the performing individual dye techniques. PMA-ddPCR and EMA-ddPCR were performed on short-term and accelerated storage samples to better understand their individual contributions to PE51-ddPCR and to gain specific insight into probiotic’s physiology during storage. As expected, both individual stain techniques overestimated viable counts compared to PC (negative biases) and had larger LOA indicating poor agreement. This is likely due to the use of suboptimal concentrations of PMA and EMA, designed to determine individual contributions to PE51, rather than as a direct comparison to PC. The data does however help visualize strain specific differences in functionality, in particular a relatively large overestimation of La-14 viable counts by PMA-ddPCR.

Despite the use of suboptimal PMA and EMA concentrations in this study, the linear analysis demonstrated that v-ddPCR at these concentrations of dye can accurately detect changes in viable cell concentrations over a 2-log dilution. This means, that while results may be skewed (higher in v-ddPCR), correlation would remain unaffected. Results however confirm initial findings of the Bland–Altman and linear analyses. Strain specific differences such as La-14 for PMA-ddPCR and Lr-32 for EMA-ddPCR add to poor correlation with PC.

Differences in performance of the single dye techniques were also detected between short-term and accelerated storage conditions. Correlation analysis and PCA showed that EMA-ddPCR and PE51-ddPCR (and PC) were closely related on short-term stored samples, while PMA-ddPCR exhibited a closer relationship with PE51-ddPCR (and PC) at accelerated conditions. These results are indicative of the manner in which the cells experience stress during storage. These findings suggest that probiotic viability during storage is subject a two-step process. Initially cells experience changes in cellular activity, potentially due to harsh processing steps and early storage. Later in storage, further loss in viability is compounded by damage to the physical cell membrane, as shown in previous research (Shu et al., 2018).

In this study PE51-ddPCR exhibited good correlation with traditional PC and showed potential as an equivalent method for enumerating FD probiotics during storage. While the 5:1 ratio of PMA to EMA performed well, this method could be improved by adjusting the ratio for strains with biases to either dye. Utilizing individual dye v-ddPCR techniques, this study identified physiological differences occurring between FD probiotics under short-term and accelerated storage conditions. These results begin to help uncover how probiotic bacteria behave during storage and suggest that loss in viability may occur in two distinct phases. While these findings support the use of PE51-ddPCR as a suitable enumeration method for FD probiotics during storage, a long term (2-year) storage stability study is underway to corroborate these findings.

## Data availability statement

The original contributions presented in the study are included in the article/Supplementary material, further inquiries can be directed to the corresponding author.

## Author contributions

AK, VF, and CW contributed to the conception and experimental design of the work. AK, PB, and GJ contributed to the acquisition of the data. AK, PT, and VF performed the statistical analysis. AK, PT, PB, KG, and VF contributed to the interpretation of the data and analyses and contributed to drafting the manuscript. All authors contributed to the article and approved the submitted version.

## Funding

This work was entirely funded by International Flavors & Fragrances, Inc. The authors declare that this study received funding from International Flavors & Fragrances, Inc. The funder was not involved in the study design, collection, analysis, interpretation of data, the writing of this article, or the decision to submit it for publication.

## Conflict of interest

AK, PMB, PT, GJ, KG, VF and CW were employed by IFF Health and Biosciences.

## Publisher’s note

All claims expressed in this article are solely those of the authors and do not necessarily represent those of their affiliated organizations, or those of the publisher, the editors and the reviewers. Any product that may be evaluated in this article, or claim that may be made by its manufacturer, is not guaranteed or endorsed by the publisher.

## Supplementary material

The Supplementary material for this article can be found online at: https://www.frontiersin.org/articles/10.3389/fmicb.2022.966264/full#supplementary-material
